# Serological Detection of Ovine Gammaherpesvirus 2 Antibodies in Dairy Farms from Southern Brazil

**DOI:** 10.3390/microorganisms12122629

**Published:** 2024-12-19

**Authors:** Selwyn Arlington Headley, Dawn Marie Grant, Juliana Torres Tomazi Fritzen, Felippe Danyel Cardoso Martins, Stefany Lia Oliveira Camilo, Eloiza Teles Caldart, Júlio Augusto Naylor Lisbôa, Amauri Alcindo Alfieri, George Cameron Russell

**Affiliations:** 1 Laboratory of Animal Pathology, Department of Preventive Veterinary Medicine, Universidade Estadual de Londrina, Londrina 86057-970, Brazil; 2 Multi-User Animal Health Laboratory (LAMSA), Department of Preventive Veterinary Medicine, Universidade Estadual de Londrina, Londrina 86057-970, Brazil; alfieri@uel.br; 3 National Institute of Science and Technology for Dairy Production Chain (INCT–LEITE), Department of Preventive Veterinary Medicine, Universidade Estadual de Londrina, Londrina 86057-970, Brazil; 4 Moredun Research Institute, Pentlands Science Park, Midlothian, Edinburgh EH26 0PZ, UKgeorge.russell@moredun.ac.uk (G.C.R.); 5 Laboratory of Animal Virology, Department of Preventive Veterinary Medicine, Universidade Estadual de Londrina, Londrina 86057-970, Brazil; jufritzen@uel.br; 6 Veelab Medicina Veterinária Diagnóstica, Londrina 86047-285, Brazil; felippew@gmail.com; 7 Large Animal Internal Medicine, Department of Veterinary Clinics, Universidade Estadual de Londrina, Londrina 86057-970, Brazil; stefanyliacamilo@hotmail.com (S.L.O.C.); janlisboa@uel.br (J.A.N.L.); 8 Laboratory of Protozoology and Parasitic Diseases, Department of Preventive Veterinary Medicine, Universidade Estadual de Londrina, Londrina 86057-970, Brazil; eloizacaldart@uel.br

**Keywords:** epidemiology, *Macavirus*, malignant catarrhal fever, serodiagnosis, spatial analysis, wild boars

## Abstract

Sheep-associated malignant catarrhal fever (SA-MCF) is a severe lymphoproliferative vascular disease of cattle that is caused by ovine gammaherpesvirus 2 (OvGHV2), which is a *Macavirus* within the *Gammaherpesvirinae* subfamily. SA-MCF occurs worldwide in several mammalian hosts. Alternatively, alcelaphine gammaherpesvirus 1 (AlGHV1) is a *Macavirus* that causes wildebeest-associated malignant catarrhal fever (MCF), which principally occurs in cattle from Africa. Previous serological assays to evaluate the presence of MCF in mammals used a competitive inhibition enzyme-linked immunosorbent assay (CI-ELISA). This CI-ELISA is based on the 15A antigenic epitope that is common to all *Macavirus* associated with the development of MCF in their respective hosts. This study evaluated an indirect MCF-specific ELISA assay based on the AlGHV1 C500 strain to detect antibodies against OvGHV2 in 43 closed dairy cattle farms from Southern Brazil. These farms are located in a region where subclinical infections by OvGHV2 have been detected in free-ranging wild boars (*Sus scrofa*). Sheep or goats were not reared at these farms or within the proximity of these farms. Risk factors associated with seropositivity to OvGHV2 were evaluated, while the possible participation of subclinically infected wild boars in the dissemination of OvGHV2 was estimated using spatial analysis. Sera from 29 dairy cows from 16 farms demonstrated sample/positive (S/P) values considered positive with this MCF-specific ELISA (cutoff S/P, 0.063). The S/P values for the positive dairy cows varied between 0.0633 and 0.2510 (mean, 0.0998; standard deviation, 0.0476). At least one cow was seropositive in 16/43 (37.2%) of these farms, with seropositivity identified in 29/367 (7.9%) of dairy cows maintained at these farms. Additionally, dairy cows raised within the intensive system had a more than threefold higher chance of being seropositive to OvGHV2 relative to those reared within the semi-intensive system. Furthermore, the spatial evaluation revealed that cows on dairy farms within a 50 km radius of the home range of subclinically infected wild boars had an increased risk of being seropositive to this assay. These findings demonstrated that the AlGHV1 C500-specific MCF ELISA can be efficiently used to monitor the occurrence of OvGHV2 in cattle. In addition, the occurrence of subclinically infected free-ranging wild boars within a radius of 50 km from susceptible cattle may be a possible risk factor for the occurrence of OvGHV2-related infections in these animals from Southern Brazil. These initial results are fundamental to understanding the epidemiology of OvGHV2-associated infections and clinical SA-MCF in mammals in Brazil.

## 1. Introduction

The ovine gammaherpesvirus 2 (OvGHV2) species *Macavirus ovinegamma* 2 belongs to the *Macavirus* genus and *Gammaherpesvirinae* subfamily [1]. *Macaviruses* known to be associated with the development of malignant catarrhal fever (MCF) in susceptible hosts are collectively referred to as the MCF virus (MCFV) complex [2,3,4]. These MCF viruses share the 15A antigenic epitope [5] and are highly conserved in genomic organization and at the sequence level [2]. Furthermore, cross-neutralization suggests two distinct serogroups of MCFVs circulating in their respective hosts: ovine/caprine and alcelaphine/hippotragine [6]. Although nine members of the *Macavirus* are officially recognized [1], only OvGHV2, alcelaphine gammaherpesvirus 1 and 2 (AlGHV1 and 2), caprine gammaherpesvirus 2 and 3, and Ibex-MCFV are currently recognized as being associated with clinical MCF [4,7,8,9,10]. However, two syndromes of MCF that are of economic and epidemiological importance worldwide are intensively studied: sheep associated MCF (SA-MCF) and wildebeest associated MCF (WA-MCF) [7,8,9,10,11,12]. Sheep are the asymptomatic hosts for OvGHV2, with clinical SA-MCF- or subclinical OvGHV2-related infections occurring in a wide range of mammalian hosts worldwide [7,8,9]. In contrast, WA-MCF is caused by AlGHV1, and wildebeest are the asymptomatic hosts, with infections occurring predominantly in cattle from Africa and in ruminants maintained in some zoological parks in the USA [7,9,11,12].

The 15A antigenic epitope, which is located on the viral glycoprotein complex of *Macavirus* [3,13], served as the basis for the development of competitive inhibition enzyme-linked immunosorbent assays (CI-ELISAs) [3,14]. However, this diagnostic assay is currently only available for use at the diagnostic laboratory of Washington State University, USA. These assays were then used in serological studies for the detection of MCFV in cervids from the USA [15,16,17,18,19] and Europe [20,21,22,23], as well as in ruminants from the USA [19,24,25], Europe [26], Turkey [27], and India [28]. Our research group standardized an immunohistochemistry (IHC) assay using the 15A-monoclonal antibody (15A-MAb) based on the 15A antigenic epitope for the detection of intralesional tissue antigens of MCFV [29]. This IHC assay was successfully used for the detection of MCFV tissue antigens in cattle [30,31,32,33,34], sheep [35], goats [36], and wild boars [37] infected with OvGHV2 in Brazil. However, since the 15A antigenic epitope is common to MCFV associated with the development of MCF in several specific hosts [3,5,14], detection using this methodology does not indicate which specific MCFV was involved in the infection. Therefore, confirmation of the type of MCFV requires the utilization of another specific diagnostic method, such as molecular assays.

Studies conducted in Brazil have confirmed clinical SA-MCF in cattle [30,38,39,40] and pigs [41], and even in a horse [42] that had no contact with the asymptomatic carrier hosts. Furthermore, similar epidemiological situations were described in bison from the USA [43,44]; while prevailing wind conditions for aerosol transmission and/or mechanical dissemination by birds [43] were used to explain the absence of sheep in one SA-MCF outbreak in bison. In contrast, there is initial evidence to suggest that subclinically infected free-ranging wild boars (*Sus scrofa*) may serve as possible bridge hosts for the dissemination of OvGHV2 in Southern Brazil in the absence of the recognized carrier host [37].

Epidemiological data suggest that SA-MCF occurs in all geographical regions of Brazil [7]. Initial analysis of the OvGHV2 glycoprotein B gene indicates that the same strain of this virus may probably be associated with SA-MCF in cattle diagnosed in different time frames and maintained in distinct biomes of Brazil [45]. Additionally, tissue antigens of an MCFV were the most frequent etiological agent (53.3%; 64/120) associated with the development of bovine respiratory disease in a study with cattle in several geographical regions of Brazil [34]. Furthermore, an MCFV was detected by IHC in the kidneys of 41.7% (48/115) of cattle with histological evidence of renal disease in Paraná State, Southern Brazil [33]. Moreover, intralesional tissue antigens of an MCFV were detected in multiple organs of two cows in the central–northern mesoregion of Paraná State, with histological evidence of OvGHV2-related infection but without the concomitant molecular detection of OvGHV2 DNA [32]. Since OvGHV2 is the only MCFV identified thus far in mammals in Brazil [7], it is reasonable to suggest that the agent is most likely OvGHV2. Nevertheless, these studies suggest that there is an elevated prevalence of an MCFV circulating in cattle from diverse geographical regions of Paraná State, Brazil. Therefore, studies must be conducted to determine which MCF is prevalent in cattle farms in Paraná State, as well as other geographical locations of Brazil.

Consequently, specific serological assays are required to evaluate and determine the presence of OvGHV2 in cattle and ruminant populations in Brazil. Excellent candidates are the indirect ELISA assays based on the OV65delB of OvGHV2 [46], the AlGHV1 C500 strain [47,48], and the AlGHV1 WC11 strain [49,50]. Although SA-MCF occurs within all geographical regions of Brazil [7], as well as in all biomes of this continental nation [45], serological evaluations were not performed to evaluate the degree of cattle exposure to this virus. Information relative to the serological profile of susceptible ruminant populations in Brazil is fundamental to understanding the epidemiology of SA-MCF in this country since subclinically infected cases of OvGHV2-related infections seem to be more common than previously documented. Accordingly, a serological investigation was conducted to evaluate the seropositivity of dairy cattle herds to antibodies of OvGHV2.

The main objectives of this study were (1) to determine if the ELISA based on the AlGHV1 C500 strain can be used as a serological assay to detect OvGHV2 antibodies in cattle; (2) to evaluate the distribution of OvGHV2 antibodies in adult dairy cattle; (3) to identify possible risk factors associated with seropositivity to OvGHV2; and (4) to determine if there is any association between the serological detection of OvGHV2 antibodies in dairy cattle and the presence of free-ranging subclinically infected wild boars within the central–eastern mesoregion of Paraná State, Southern Brazil.

## 2. Materials and Methods

### 2.1. Study Location, Dairy Farms, and Sampling

This study was conducted in municipalities located within the high-yield milk-producing region of the state of Paraná, Southern Brazil. These dairy farms (*n* = 43) are located within the municipalities of Arapoti, Carambeí, Castro, Jaguariaíva, and Palmeira in the central–eastern mesoregion of this state. The state of Paraná was the second largest producer (4,472,406 liters) of milk in Brazil in 2022, with the municipality of Castro being the home to the largest milk producers (426,595 liters) within the state of Paraná and the second largest milk-producing region of Brazil [51]. Paraná State is located within the Atlantic Forest biome of Brazil.

Dairy farms within this region use several technologies to maximize milk production and consist exclusively of cows from the Holstein and Jersey breeds of cattle. Sheep or goats are not reared concomitantly with dairy cows on these farms nor within the proximity of these establishments. However, to assess the possible influence of sheep on the occurrence of SA-MCF, the sheep/cattle ratio (SCR) within each municipality was estimated. The SCR was used since this index may be used to predict the occurrence of SA-MCF in specific geographical regions of Brazil [52]. The SCR was determined by using the officially recorded populations of sheep and cattle reared in each municipality in 2019 [53].

Most of these farms (40/43; 90%) are closed units, in which animals are not introduced or acquired, with the exception being Farms #23, 24, and 30. Furthermore, cows were milked twice daily at most farms (31/43; 72.1%), while milking at the other farms (12/43; 27.9%) occurred thrice daily. Most of these farms (28/43; 65.1%) adopt the intensive rearing system of dairy cattle. Additionally, these dairy farms are located within the central–northern mesoregion of Paraná State, where OvGHV2 was detected in subclinically infected wild boars [37,54].

At least eight samples were collected randomly from adult cows at each farm between October and December 2019. Blood samples were collected from the caudal vein of all cows using a vacuum hypodermic needle (30 × 0.8 mm) in tubes without anticoagulant. The coagulated blood samples were then centrifuged for 10 min (1500× *g*) to separate blood serum; the serum obtained was maintained at −80 °C until it was used in serological assays.

#### Definitions

Seropositive dairy farm: A dairy farm was considered to be positive for OvGHV2 after the serological detection of MCFV-specific antibodies in at least one cow from a particular farm.

Intensive management of dairy cows: The cows were reared exclusively within enclosed areas (such as compost barns or free stalls) during their adult lives and even during the dry period.

Semi-intensive management of dairy cows: The adult cows were maintained partially within enclosed areas and spent part of their adult lives in grass paddocks.

Extensive management of dairy cows: The adult dairy cows were not maintained within enclosed areas.

These definitions do not reflect the rearing of dairy heifers at these farms, who may have spent some part of their preadult lives within grass paddocks. Consequently, irrespective of the type of management system, all cows may have had access, though limited, to grass paddocks.

### 2.2. Serological Detection of OvGHV2 Antibodies by Indirect ELISA

The serological detection of OvGHV2 antibodies was performed using a modified MCF-specific ELISA that contained AlGHV1 C500-positive antigen and BT-negative antigen [47,48]. These antigens and control serum samples were generously provided by an international cooperation between DMVP-Universidade Estadual de Londrina, Brazil, and the Moredun Research Institute, UK.

For the analysis of antibody binding to MCF antigens, the positive and negative antigens were diluted to 5 μg/mL in 0.1 M of carbonate buffer with a pH of 9.6. Pairs of adjacent rows of 96-well microtitre plates (Nunc Immuno Plate F96 Maxisorp, Thermo Fisher Scientific, Waltham, MA, USA) were then coated with positive and negative antigens, respectively, and incubated overnight at 4 °C. Wells containing bound antigen were washed 6 times with PBS/0.05% Tween20 (PBS-Tween) before blocking with 4% filtered non-fat dried milk/PBS for 1 h. The plates were then washed as described above, and serum samples and positive and negative controls, which were diluted 1:1000 using 2% filtered non-fat dried milk in PBS-Tween, were applied to the positive and negative antigen wells in duplicate. One hour later, the plates were washed again. Bound antibodies were detected using rabbit α bovine IgG-HRP conjugate (A5295, Sigma; Saint Louis, MO, USA) that was diluted 1:1000 in PBS-Tween and incubated for 1 h. The plates were then washed, and TMB-peroxidase substrate (Life Technologies, Carlsbad, CA, USA) was applied for five minutes. The reaction was stopped by the addition of 0.1 M of HCl, after which the plates were read at 450 nm in a plate reader (Biotek Elx800 Microplate Reader, Winooski, VT, USA).

The ELISA values for each serum sample were calculated as the difference between the average absorbance values at 450 nm (OD450) for the positive antigen (C500 CHAPS) minus the average OD values for the negative antigen (BT CHAPS). The determination of the sample to positive (S/P) values, means, and sample standard deviation (SD) was performed in Microsoft Excel. All samples with S/P values greater than 0.063 were considered to be positive [48].

### 2.3. Geographical Locations of Dairy Farms and OvGHV2-Infected Free-Ranging Wild Boars

The geographical location of each farm was plotted on a map generated by the QGIS software, version 3.18 [55]. Additionally, the locations of these farms were compared with the known locations where free-ranging wild boars subclinically infected by OvGHV2 were detected [37,54]. All components used in this map were open-source and open-data (https://github.com/qgis/QGIS/tree/master/images/svg, 20 August 2024). The determination of municipal and other boundaries was performed using official public data of the Government of Brazil (https://geoftp.ibge.gov.br/cartas_e_mapas/bases_cartograficas_continuas/bc250/versao2021/post_gis/bc250_2021_11_18.zip, 20 August 2024), and the boundaries were used as a background base layer. Information for the conservation unit system was derived from official data from the government of Brazil (https://antigo.mma.gov.br/biomas.html 20 August 2024).

### 2.4. Spatial Evaluation of the Possible Role of Subclinically Infected Free-Ranging Wild Boars as Disseminators of OvGHV2

Since these dairy farms are enclosed units, and sheep were not reared within these properties nor the adjacent areas, the possible contact with sheep was negligible. However, since asymptomatic OvGHV2-infected free-ranging wild boars were diagnosed within this geographical region [37,54], we tested the hypothesis that these animals had a role in the dissemination of this virus to dairy cows maintained at these farms.

To identify the risk of cattle becoming seropositive following contact with asymptomatic free-ranging wild boars, a statistical spatial analysis was conducted to compare the distance between positive and negative dairy farms in relation to the wild boar home range, where subclinically infected wild boars were captured [37,54]. To this end, a home range for wild boars was delimited by a buffer formed by a radius of 50 km around the capture site of all subclinically infected wild boars. This distance was based on the estimated home range for wild boars, which can vary between 30 and 87 km^2^ [56]; the buffer zone was established with the QGIS program, Version 2.18.14. A comparison of the occurrence of dairy farms within and outside this buffer area was performed using the chi-squared test. The significance was set at *p* < 0.05. The representative map was then created with the QGIS program.

### 2.5. Statistical Analyses

When possible, the results were presented with descriptive statistics. The determination of seropositivity and confidence intervals were calculated based on the available data. Statistical analyses were conducted separately to detect possible risk factors associated with seropositivity to OvGHV2 antibodies at the dairy farms (*n* = 43) and in all cows (*n* = 367) evaluated from these farms. The chi-squared test or Fisher’s test within SigmaPlot for Windows, Version 13.0 (Systat Software Inc., San Jose, CA, USA) was used to determine the possible factors associated with seropositivity to OvGHV2 at the farms and in dairy cows. Additionally, any variable with *p*  ≤  0.20 was subjected to multinomial logistic regression to determine the possible risk or protective factors. The multinominal analyses were conducted with SPSS software, version 27 (International Business Machines Corporation, Armonk, NY, USA), with a 95% confidence level.

## 3. Results

### 3.1. Distribution of S/P Values of C500-AlGHV1 MCF ELISA in Dairy Cows in Southern Brazil

**Figure 1:**
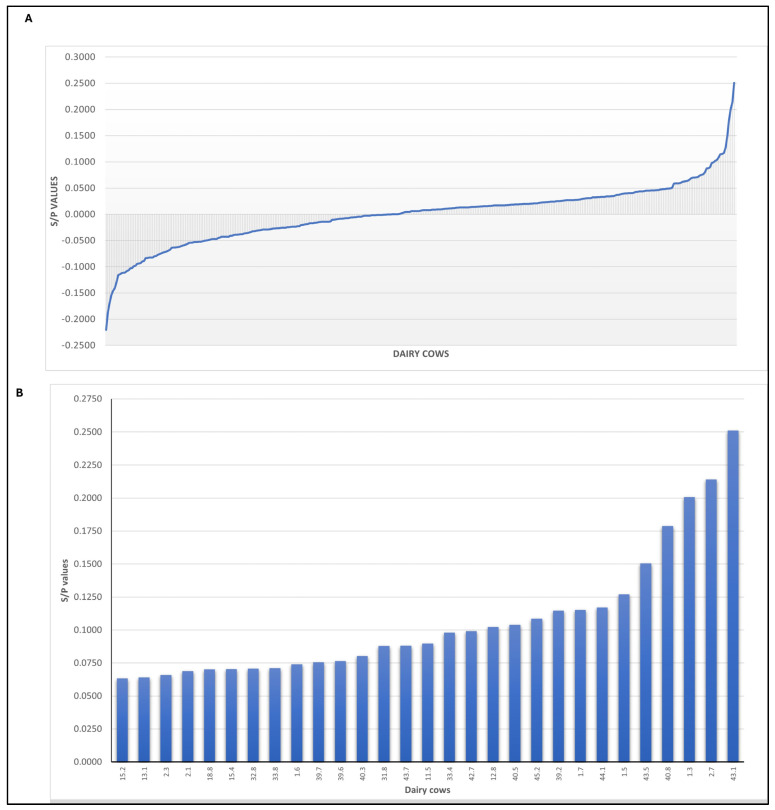
The distribution of the MCF ELISA S/P values identified in dairy cows in Southern Brazil. Panel (**A**) shows the S/P values of all cows evaluated, while panel (**B**) focuses on the S/P values of the seropositive cows. The identification of each cow at the respective dairy farm is provided; there is a 0.063 cutoff for this assay.

The S/P values of the 367 cows evaluated with the MCF ELISA varied from −0.220 to 0.251 (Figure 1; Supplemental Table S1). Additionally, 29 cows had S/P values greater than 0.063 and were considered positive with the C500-AlGHV1 ELISA. The S/P values for the positive cows ranged from 0.0633 to 0.2510 (mean, 0.0988; SD, 0.0476).

The range of the MCF S/P values for the positive cows within each municipality is provided in Table 1; seropositivity to the MCF ELISA was detected in dairy cows reared in four of the municipalities evaluated. The highest positive S/P value (Table 1) was detected in a cow from the municipality of Castro (S/P; 0.251), with the lowest being in another from Carambeí (S/P; 0.0633). The S/P values of the 338 seronegative cows ranged from −0.2204 to 0.0623. Eight cattle on a single farm in the municipality of Jaguariaíva were tested. None tested positive, and the S/P values varied between −0.051 and 0.034.

The distribution of the S/P values of the MCF ELISA assay observed in the dairy cows during this study is illustrated in Figure 1. The S/P values for all 367 dairy cows evaluated are given in Figure 1A, while the dairy cows with positive S/P values are shown in Figure 1B.

### 3.2. Serological Detection of OvGHV2 Antibodies in Dairy Farms and Cows in Southern Brazil

The distribution of positive dairy farms and OvGHV2-seropositive cows are presented in Table 2. There was positive seroreactivity to OvGHV2 among dairy cows maintained in most of the municipalities investigated (80%; 4/5), with the exception being the municipality of Jaguariaíva, where only a single farm was tested. When the occurrence of seropositivity between dairy farms from the municipalities was compared, the frequency of OvGHV2 seropositivity (Table 2) was higher at farms within the municipalities of Carambeí (50%; 5/10) and Palmeira (40%; 4/10), while the lowest seropositivity was detected in farms within the municipality of Arapoti (28.5%; 2/7). Nonetheless, 37.2% (16/43) of all the farms investigated contained at least one cow that was seropositive for OvGHV2.

When all cows evaluated were considered, the occurrence of seropositivity to OvGHV2 in dairy cows varied between 7.5% (9/120) in animals within the municipality of Castro and 12.3% (7/57) in dairy cows reared within the municipality of Arapoti (Table 2). Moreover, the average seropositivity to OvGHV2 identified in dairy cows was 7.9% (29/367).

### 3.3. Factors Associated with Seropositivity to OvGHV2 Antibodies in Dairy Cows and Farms from Southern Brazil

Some factors that could have influenced the occurrence of OvGHV2-seropositive dairy farms and cows are presented in Table 3. The frequency of milking (twice or thrice daily) was not associated with seropositivity to OvGHV2 when the dairy farms or cows were evaluated. Furthermore, the type of management system (intensive or semi-intensive) did not influence the occurrence of seropositivity to OvGHV2 between the dairy farms but was associated with seropositivity when the dairy cows were evaluated. A significant statistical difference (*p* = 0.030) was detected when the frequency of seropositive dairy cows reared intensively (10.5%) was compared with cows reared under semi-intensive management (2.9%).

Additionally, the bivariate analysis demonstrated that dairy cows maintained within the intensive rearing system had a 3.95x higher chance (CI, 95%; 1.17–13.34) of being seropositive to OvGHV2 antibodies, relative to dairy cows reared within the semi-intensive system (Table 3). The SCR (Supplemental Table S2) for the municipalities with seropositive cows was very low (Arapoti, 0.05; Carambeí, 0.03; Castro, 0.12; and Palmeira, 0.32), while the relationship between the number of goats and cattle reared within each municipality varied from 0 to 0.1.

### 3.4. Geographical Localization of Farms with Seropositive Cows to OvGHV2

**Figure 2:**
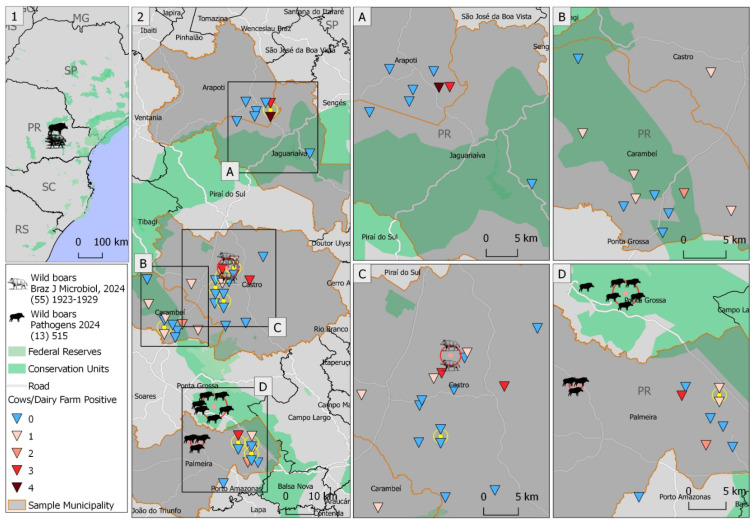
Geographical locations of the dairy farms and subclinically infected wild boars within the municipalities of the central–eastern mesoregion of Paraná State, Southern Brazil. The overview of the study location is provided (**1**) with details of the positive and negative dairy herds and OvGHV2 positive free-ranging wild boars (**2**). Legend: (**A**) Municipalities of Arapoti and Jaguariaíva; (**B**) Carambeí; (**C**) Castro; and (**D**) Palmeira [37,54].

The geographical locations of the 43 dairy farms evaluated during this study, the five locations where free-ranging wild boars subclinically infected by OvGHV2 were captured [37,54], as well as the number of positive dairy cows/farms are presented in Figure 2. The study area is shown in Figure 2(1), while the geographical locations of the dairy farms and subclinically infected wild boars are illustrated in Figure 2(2). The largest number of seropositive cows (*n* = 4) was detected in one dairy farm in the municipality of Arapoti (Figure 2A), followed by the detection of three seropositive cows on farms within the municipalities of Arapoti (Figure 2A), Castro (Figure 2C), and Palmeira (Figure 2D).

Most of the positive dairy farms were located within the municipalities of Carambeí (*n* = 5), Castro (*n* = 5), and Palmeira (*n* = 4). Interestingly, subclinically infected free-ranging wild boars were captured within 5 km of the positive farms within the municipality of Castro (Figure 2C) and within approximately 10 km of the positive farms within Palmeira (Figure 2D). Although subclinically infected wild boars were not detected within the municipalities of Arapoti, Jaguariaíva, and Carambeí (Figure 2A,B), there are constant sightings of free-ranging wild boars within these municipalities [57,58].

### 3.5. Possible Relationship Between Subclinically Infected Free-Ranging Wild Boars and Seropositive Dairy Farms

**Figure 3:**
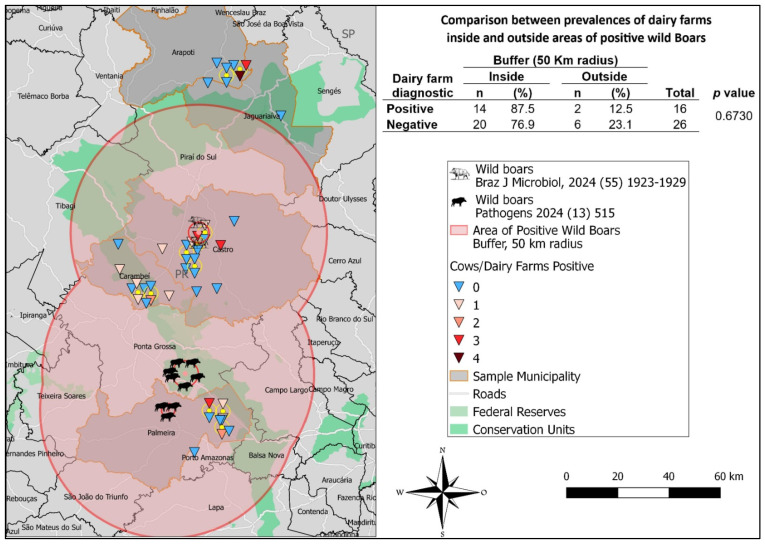
Spatial relationship between dairy farms and subclinically infected wild boars. The buffer zone for the home area of wild boars is shown (50 km radius). The statistical chi-squared analysis of the relationship between dairy farms and subclinically infected wild boars is provided in the contingency table [37,54].

When all the seropositive dairy farms investigated using spatial analysis were evaluated (Figure 3), 87.5% (14/16; CI 95%: 72.9%–100%) of these were within the wild boar home area (50 km buffer). Additionally, 76.9% (20/26; CI 95%: 59.3%–92.7%) of the negative dairy farms were also within this area. Consequently, there was no significant statistical difference (*p* = 0.673) between the proportion of positive and negative dairy farms within the wild boar home area. However, the risk of having seropositive dairy farms within the buffer zone was 3.3 times greater than dairy farms located outside the buffer zone (Fisher’s test: *p =* 0.0851; relative risk = 3.2941; 95% CI, 0.8478–12.7992). Finally, it is worth mentioning that only farms within the municipalities of Arapoti and Jaguariaíva clustered outside the buffer zone, where two positive dairy farms were located within the municipality of Arapoti (Figure 3).

## 4. Discussion

The results of this study demonstrated that 37.2% (16/43) of the dairy herds investigated and 7.9% (29/367) of the cows maintained on these farms contained antibodies against OvGHV2. The number of positive cattle samples ranged from one to four within each farm group of eight to ten samples. Additionally, seropositive cows were identified in farms from all municipalities evaluated, except Jaguariaíva, where only one group of eight cattle was tested. Therefore, these results suggest that OvGHV2 infection may be more widely distributed in dairy cattle herds than previously documented. Additionally, this is the first study conducted in Brazil, and South America, to evaluate the seropositivity of animals to any *Macavirus*. Consequently, the results described herein are novel findings that will provide fundamental epidemiological data for the understanding of SA-MCF in this continental nation. The epidemiology of SA-MCF in Brazil has several gray areas, particularly with respect to the presence and/or absence of reservoir hosts associated with clinical outbreaks of MCF or in subclinical OvGHV2-induced infections.

This is particularly true in cases of subclinical infections due to OvGHV2 in cattle in Brazil. Our research group has frequently identified subclinical infections, predominantly in cattle, by demonstrating histological evidence of OvGHV2-associated lesions in this geographical region of Brazil [30,31,32,59]. Additionally, OvGHV2 DNA was identified in a goat [36] and bovine fetuses [60] without histological evidence of disease. Subclinical infections were also confirmed in free-ranging wild boars [37,54], which may be a potential bridge-host for this virus [37]. Furthermore, OvGHV2 DNA was identified in water buffalos [61], as well as in two horses that died after presenting an acute neurological syndrome and fetal tissues of two sheep without histopathological evidence of subclinical infections (Headley, S.A.; personal communication [62]). In some cases of subclinical infections, neither sheep nor goats were reared alongside the cattle [30,32] or within the proximity [31] of the affected cattle. In other geographical regions and biomes of Brazil, there are also reports of clinical SA-MCF in cattle [38,40] and pigs [41] that had no prior contact with an asymptomatic carrier host. Consequently, there is accumulating evidence to suggest that the epidemiology and pathogenesis associated with clinical SA-MCF or subclinical OvGHV2-related infections in mammals in Brazil may be somewhat different than those described elsewhere.

The indirect ELISA used in this study is based on the attenuated strain AlGHV1 C500 [47], which is a candidate for the development of a vaccine against MCF [63]. This assay can be used to evaluate the seroconversion of MCFV affecting ruminant populations, including cattle, bison, wildebeest, and deer [46], and is an efficient diagnostic assay [48]. Similar serological assays performed in Japan based on the AlGHV1 WC-11 and using complement fixation reported seropositivity to OvGHV2 in small ruminants [49,64] and cows [64].

Although serological evaluations to detect AlGHV1 infection are scarce [12], a few investigations have been published. Serological evaluations performed in Tanzania with the C500 AlGHV1 ELISA identified AlGHV1 antibodies in 1.1% (4/362) of cattle in the Simanjiro Wildlife Dispersal Area [65], 5% (10/200) of cattle in the Tarangire District, and all (*n* = 13) the wildebeest evaluated in the Ngorongoro Crater [66]. Additionally, excellent correlations were obtained when the specificity and sensitivity between the indirect AlGHV1 WC-11 ELISA and the CI-ELISA based on the 15A epitope of *Macavirus* were compared [50].

### 4.1. Indirect C500-AlGHV1 ELISA Can Be Used to Detect OvGHV2 Antibodies in Cattle

The S/P values of all seropositive cows varied between 0.0631 and 0.251, while the S/P values for seronegative cows ranged between −0.220 and 00630. As indicated previously, 7.9% (29/367) of the dairy cows evaluated demonstrated antibodies to OvGHV2 in the absence of any clinical signs of MCF. These findings confirmed that this AlGHV1-based MCF-specific ELISA can be used to detect OvGHV2 antibodies in cattle and provide an efficient tool to investigate the exposure of ruminants to this virus. Additionally, it was recently demonstrated that the C500 ELISA assay has a 93% sensitivity and 98% specificity for the detection of OvGHV2 antibodies within serum samples of cattle [48]. These serological results support previous IHC findings that demonstrated the occurrence of an MCFV, likely OvGHV2, in cattle in Brazil with pulmonary [34] and renal [33] lesions using the intralesional detection of tissue antigens within the lungs and kidneys, respectively. It must be highlighted that in both studies [33,34], all cattle investigated had no clinical diagnosis of SA-MCF, despite having lesions containing virus antigen. This correlates well with the current study, in which all cattle had no prior diagnosis of clinical SA-MCF. A previous serological study using the CI-ELISA, which is based on the 15A epitope of *Macavirus*, reported seropositivity of 40% among dairy cattle on a farm in the USA with previous outbreaks of SA-MCF [24] and 15% seropositivity among cattle reared alongside sheep in Turkey [27]. The results of the current study are of great importance not only due to the absence of any obvious SA-MCF in the evaluated cattle population but also due to the detection of OvGHV2 antibodies in dairy cattle without documented prior contact with the asymptomatic carrier host (see below). Additionally, these initial findings are of greater concern when the rate of seropositivity within the dairy herds is considered since 29% of these farms contained at least one cow with demonstrable antibodies to OvGHV2. In addition, since there was no recent introduction of cows into these farms, it is possible that OvGHV2 could have been endemic within these establishments, although it is unclear how the virus could spread within these herds. Consequently, these results suggest that the occurrence of OvGHV2 in dairy herds from Southern Brazil may be more widespread than previously suspected.

In Brazil, subclinical [30,31,32,39,45] or neurological manifestations [29,67,68] of OvGHV2-associated infections in cattle seem to be more frequent than outbreaks of clinical SA-MCF with the classical head-and-eye presentation. Subclinical infections due to OvGHV2 have been diagnosed in cattle [14,24,26,69], buffalo [61,69,70], and bison [71,72] from diverse geographical locations worldwide and were also reported in WA-MCF [65]. We had previously estimated that the cost associated with mortality due to clinical SA-MCF in cattle from Brazil may vary between USD 3.2 billion and USD 4.8 billion/year [7]. However, our current findings may suggest that the actual costs may be more than the estimated value since infections by OvGHV2 may be more disseminated and widespread in cattle herds than previously demonstrated. These associated costs due to OvGHV2 infections will also be increased if the effects of pulmonary impairment are included, due to the association of OvGHV2 with the development of pulmonary disease in cattle [73]. Accordingly, detailed serological investigations are being implemented to evaluate the seropositivity of OvGHV2 in dairy and beef cattle in different geographical locations and biomes of Brazil.

As previously mentioned, serological evaluations with the C500-AlGHV1 ELISA detected antibodies to AlGHV1 in 1.1% (4/362) [65] and 5% (10/200) [66] in cattle in distinct regions of Tanzania. Additionally, serology using the WC11 ELISA revealed that 3 of 197 samples (1.5%) were above the assay cutoff [50]. These results are quite different from the 7.9% observed in dairy cows during the current study. Although the exact reason for the differences in seropositivity between these studies is unknown, the effects of the differences in the systems of cattle rearing and breeding, geographical locations, and environmental pressures between these two distinct geographical locations [45] and distinct pathogenic effects of these two agents may be possible explanations. The elevated level of subclinical infections in cattle relative to other geographical areas, coupled with the detection in wild boars, may suggest that the strains of OvGHV2 circulating in Brazil seem to be distinct from those identified in other geographical regions, with possible horizontal circulation among cattle, and even wild boars. Nevertheless, these initial results suggest that the C500 AlGHV1 ELISA can be used successfully to demonstrate antibodies to OvGHV2 in cattle in South America, as well as antibodies to AlGHV1 in cattle in Africa. Therefore, this assay may be an important tool for the serological evaluation of MCF viruses in cattle worldwide.

### 4.2. Dairy Cows Maintained at Farms Within the Home Range of Subclinically Infected Free-Ranging Wild Boars Were at Elevated Risk of Being Seropositive to OvGHV2

The spatial analysis demonstrated that cows maintained at dairy farms within the home range associated with subclinically infected free-ranging wild boars had a 3.2 times increased risk of being seropositive for OvGHV2. Consequently, these results imply that free-ranging wild boars may participate in the dissemination of OvGHV2, possibly acting as bridge hosts, as previously suggested [37]. As far as the authors are aware, this is the first study to statistically demonstrate a potential association between subclinically infected wild boars and the dissemination of OvGHV2. These results are exciting and may be a possible explanation for outbreaks of SA-MCF [38,39,40] or infections associated with OvGHV2 [30,32] in diverse geographical regions of Brazil without the presence of sheep at the affected establishments. Interestingly, OvGHV2 was detected in sheep on several farms within the Cerrado biome of Brazil, where sheep and cattle were reared simultaneously, but without any history of MCF in cattle [74]. The possibility of wild boars participating in the epidemiology of subclinical OvGHV2-related infections and clinical SA-MCF is a novel finding that may be the missing key to understanding the dissemination of this virus. However, additional studies must be conducted to identify this virus in free-ranging wild boars from diverse geographical regions and biomes of continental Brazil.

Nevertheless, it must be highlighted that the two dairy farms with four seropositive cows within the municipality of Arapoti and the single farm investigated in the municipality of Jaguariaíva with seronegative cows were all located outside the 50 km estimated wild boar home area used in this study. However, the municipality of Castro, where subclinically infected wild boars were identified [37], is located approximately 75 km away from Arapoti, while the home range for free-ranging wild boars varies between 30 and 87 km^2^ [56]. Additionally, wild boars can roam between 76 km (during the resting phase) and 310 km during the dispersal phase [75]. Moreover, wild boar activity is frequent within the northern central mesoregion of Paraná State [57]. Therefore, it is plausible to suggest that free-ranging wild boars could have been associated with the dissemination of OvGHV2 to dairy cows within the municipality of Arapoti, considering the absence of sheep and goats on these farms and the estimated 0.05 SCR within this municipality (Supplemental Table S2).

### 4.3. Dairy Cows Reared Within the Intensive Management System Were More Likely to Be Seropositive for OvGHV2

During this study, the possibility of dairy cows reared intensively being seropositive to OvGHV2 was 3.95 times (95% CI, 1.17–13.34) higher than in dairy cows reared with semi-extensive management, suggesting that the intensive management systems favored the occurrence of OvGHV2 and may be a possible risk factor for the occurrence of this virus in dairy cattle. In Brazil, dairy cows reared within the intensive management system are maintained within enclosed areas, thereby reducing possible contact with other animals. However, this production system favors the transmission and dissemination of contagious diseases due to animal density [76]. This may imply intense cow-to-cow infection transmission, which is not consistent with the known epidemiology of OvGHV2 [7,9,10,11] since cows are considered terminal hosts in SA-MCF. Nevertheless, the active horizontal transmission of OvGHV2 among water buffalo was suggested to explain the elevated occurrence of nasal infections in a buffalo herd in Switzerland that had no contact with sheep [70]. Similarly, possible bison-to-bison transmission was suspected in an outbreak of SA-MCF in which bison calves infected with OvGHV2 had no known contact with sheep [44]. Furthermore, most of the dairy farms investigated are traditionally closed, commercially based establishments; there is no contact between sheep or goats and the dairy cows maintained at these farms, and the estimated SCR within these municipalities is relatively low. Elevated SCR may be associated with the occurrence of SA-MCF in specific geographical regions of Brazil [52]. It is worth highlighting that the lifecycle of the dairy cows at these closed establishments begins and ends at the same farm. Therefore, the occurrence of possible horizontal transmission of OvGHV2 between intensively reared dairy cows cannot be totally excluded in the current case without further investigation.

Susceptible mammals are normally infected with OvGHV2 following contact with infectious material from an asymptomatic host [8,9,10,11]. The participation of subclinically infected free-roaming wild boars as bridge hosts in the dissemination of OvGHV2 was proposed [37]. Previously, infections by OvGHV2 in bison without contact with sheep have been associated with airborne spread or mechanical transmission by birds [43]. In this case, the dairy cows reared intensively were more likely to be infected while they were calves and not during their adult milk-producing lives because as calves, they spent the early part of their lives in grass paddocks, where the chances of being infected due to incidental contact with an infected host [37] are higher. Free-ranging wild boars are common within the central–northern mesoregion of Paraná State [57,58], and it was estimated that these animals can travel at least 6 km daily [37]. Furthermore, several dairy farms with seropositive cows were located less than 5 km from regions where subclinically infected wild boars were diagnosed and within the home range of these wild boars. Moreover, the boundaries of most farms are fenced, which can be a potential route of entry for curious wild boars on their quest for available foodstuffs [58], thereby increasing the risk of incidental contact with dairy cattle maintained on grass paddocks independent of the system of dairy cattle production. Since neither sheep nor goats were reared close to these dairy farms, contact with the dairy cows was impossible. The absence of sheep (and goats) within proximity of these farms was supported by the low SCR detected in these municipalities (Supplemental Table S2). Alternatively, elevated SCR associated with cultural farming practices may be one of the factors that contributed to the elevated frequency of SA-MCF outbreaks in cattle reared within municipalities of the Pampa biome of the state of Rio Grande do Sul, Brazil [52]. Consequently, the participation of sheep and goats in the dissemination of OvGHV2 at these dairy farms was not considered responsible for the levels of seropositivity identified during this study. Accordingly, the role of other animals in the epidemiology of OvGHV2, as well as other forms of dissemination of OvGHV2, must be considered.

Additionally, all dairy cows that were serologically positive for OvGHV2 antibodies at these dairy farms had no clinical demonstration of any disease syndrome and can arguably be considered asymptomatic animals. This pattern is consistent with the diagnosis of subclinical OvGHV2 infections in cattle in this geographical region of Brazil (as described above). Although other MCFVs have not been identified in mammals in this continental nation, there is evidence of the circulation of an undiagnosed *Macavirus* in cattle in Southern Brazil [32,60]. Therefore, understanding the epidemiology of clinical SA-MCF or subclinical OvGHV2 infections in mammalian populations within the various biomes of Brazil will be challenging.

### 4.4. Study Limitations and Future Perspectives

The main setback in this study was the collection of serum samples from only one dairy farm within the municipality of Jaguariaíva, with the non-detection of seropositive cows from this specific region. Sampling from only one farm occurred due to logistic difficulties in acquiring adequate samples from more dairy farms within this municipality. Consequently, a more uniform sample collection from the dairy farms within this mesoregion would have provided a better overview of the seropositivity to OvGHV2 within this specific region of Brazil. Nevertheless, the main objective of this study was demonstrated, i.e., the utilization of the C500 MCF-specific ELISA as an epidemiological tool to monitor OvGHV2. This diagnostic strategy will be of fundamental importance in understanding the epidemiology of SA-MCF and OvGHV2-related infections in animals from Brazil. Nevertheless, studies are being implemented to evaluate the occurrence of OvGHV2 in dairy and beef cattle herds in several geographical regions of Brazil and the seropositivity of OvGHV2 in areas where cattle and sheep are reared simultaneously.

Finally, due to the high level of subclinical infection detected here and the initial evidence supporting the possible involvement of free-ranging wild boars in the epidemiology of SA-MCF in Brazil, detailed genetic comparisons between the positive samples derived from cattle and wild boars should be performed by sequencing multiple viral loci or the whole virus genome when possible.

## 5. Conclusions

A serological assay using an MCF-specific ELISA revealed that 37.2% (16/43) of the dairy farms evaluated contained at least one dairy cow that was seropositive to OvGHV2 antibodies. Additionally, 7.9% (29/367) of all dairy cows tested were seropositive to OvGHV2. These results demonstrate that the C500 AlGHV1 ELISA assay can be used to evaluate the serological status of cattle relative to exposure to OvGHV2. Furthermore, dairy cows reared within the intensive management system demonstrated a greater risk of being seropositive to OvGHV2. In addition, cows maintained on dairy farms that were located within the home range of subclinically infected free-ranging wild boars were more likely to be seropositive to this virus. These findings suggest that free-ranging wild boars may have a role in the dissemination of OvGHV2 in geographical regions of Brazil where sheep are not reared close to cattle.

## Figures and Tables

**Table 1:** Range of sample to positive (S/P) values of the C500-AlGHV1 ELISA for seropositive cows within each municipality.

Municipalities	S/P ELISA Values
Arapoti	0.0659–0.2140
Carambeí	0.0633–0.1023
Castro	0.0880–0.2510
Palmeira	0.0711–0.1146

**Table 2:** Occurrence of positive dairy farms and seroreactive cows to OvGHV2 antibodies within municipalities from the central–eastern mesoregion of Paraná State, Southern Brazil.

Municipalities	N^o^ Tested	N^o^ Positive	Frequency (%)	95% CI
*Farms*				
Arapoti	7	2	28.5	3.7–70.9
Carambeí	10	5	50	18.7–81.3
Castro	15	5	33.3	11.8–61.6
Jaguariaíva	1	0	0	-
Palmeira	10	4	40	12.2–73.8
**Total**	**43**	**16**	**37.2**	**22.9–53.3**
*Cows*				
Arapoti	57	7	12.3	5.1–23.7
Carambeí	102	6	5.9	2.2–12.4
Castro	120	9	7.5	3.5–13.8
Jaguariaíva	8	0	0	
Palmeira	80	7	8.8	3.6–17.2
**Total**	**367**	**29**	**7.9**	**5.6–11.1**

**Table 3:** Possible risk factors associated with the occurrence of seropositive farms and seropositive dairy cows to OvGHV2 antibodies.

Factors Evaluated	Positive		Negative		*p*
	N^o^	%	N^o^	%	
*Farms*					
Milking frequency					
Twice daily	12	38.7	19	61.3	1
Thrice daily	4	33.3	8	66.7	
Type of management					
Intensive	13	46.4	15	53.6	0.279
Semi-intensive	3	23.1	10	76.9	
*Cows*					
Milking frequency					
Twice daily	23	9.2	226	90.8	0.241
Thrice daily	6	5.1	112	84.9	
Type of management					
Intensive	23	10.5	221	89.5	0.030
Semi-intensive	6	2.9	101	97.2	
Multivariate analysis					
Factor	OR	95% CI	Coefficient	SE	*p*
Intensive dairy management	3.95	1.17–13.34	1.3745	0.6210	0.0269

## Data Availability

The original contributions presented in the study are included in the article/Supplementary Material, further inquiries can be directed to the corresponding author.

## Supplementary Materials

The following supporting information can be downloaded at https://www.mdpi.com/article/10.3390/microorganisms12122629/s1. Supplemental Table S1. SP values for MCF ELISA. Supplemental Table S2. The relationship between the populations of heads of cattle, sheep, and goats reared within specific municipalities of the Central-eastern mesoregion of Paraná state, Brazil in 2019.
