# Supplementary material for: Serological Detection of Ovine Gammaherpesvirus 2 Antibodies in Dairy Farms from Southern Brazil
Source: Microorganisms. 2024 Dec 19;12(12):2629. doi: 10.3390/microorganisms12122629 (PMC11676213; doi:10.3390/microorganisms12122629)
Supplement: Supplementary file 1 [file microorganisms-12-02629-s001.zip › microorganisms-3234370-supplementary.pdf]

*Article*

# Serological Detection of Ovine Gammaherpesvirus 2 in Dairy Farms from Southern Brazil

**Selwyn Arlington Hedley, [1,2,3]* Dawn Marie Grant, [4] Juliana Torres Tomazi Fritzen, [5] Felippe Danyel Cardoso Martins, [6] Stefany Lia Oliveira Camilo, [7] Eloiza Teles Caldart, [8] Júlio Augusto Naylor Lisboa, [7] Amauri Alcindo Alfieri [2,3,5] and George Cameron Russell [4].**

1 Laboratory of Animal Pathology, Department of Preventive Veterinary Medicine, Universidade Estadual de Londrina, Londrina 86057-970, Brazil
2 Multi-User Animal Health Laboratory (LAMSA), Department of Preventive Veterinary Medicine,
Universidade Estadual de Londrina, Londrina 86057-970, Brazil; alfieri@uel.br
3 National Institute of Science and Technology for Dairy Production Chain (INCT–LEITE), Department of Preventive Veterinary Medicine, Universidade Estadual de Londrina, Londrina 86057-970, Brazil
4 Moredun Research Institute, Pentlands Science Park, Midlothian, Edinburgh EH26 0PZ, UK;
george.russell@moredun.ac.uk (G.C.B.)
5 Laboratory of Animal Virology, Department of Preventive Veterinary Medicine, Universidade Estadual de Londrina, Londrina 86057-970, Brazil; jufritzen@uel.br
6 Veelab Medicina Veterinária Diagnóstica, Londrina 86047-285, Brazil; felippew@gmail.com
7 Large Animal Internal Medicine, Department of Veterinary Clinics, Universidade Estadual de Londrina, Londrina 86057-970, Brazil;
stefanyliacamilo@hotmail.com (S.L.O.C.); janlisboa@uel.br (J.A.N.L.)
8 Laboratory of Protozoology and Parasitic Diseases, Department of Preventive Veterinary Medicine,
Universidade Estadual de Londrina, Londrina 86057-970, Brazil; eloizacaldart@uel.br
* Correspondence: selwyn.headley@uel.br; Tel.: +55-43-3371-4766

Supplemental Table S1 SP values for MCF ELISA

| nº animal | S/P (sample/positive) |
|---|---|
| 1.1 | 0.019622362 |
| 1.2 | 0.047019622 |
| 1.3 | 0.20066642 |
| 1.4 | 0.005923732 |
| 1.5 | 0.126990004 |
| 1.6 | 0.074046649 |
| 1.7 | 0.11514254 |
| 1.10 | -0.106920302 |
| 2.1 | 0.068863384 |
| 2.2 | -0.153623188 |
| 2.3 | 0.065901518 |
| 2.4 | -0.129468599 |
| 2.5 | -0.082562014 |
| 2.6 | -0.028985507 |
| 2.7 | 0.213994817 |
| 2.8 | 0.062315513 |
| 2.10 | -0.05410628 |
| 3.1 | 0.023324695 |
| 3.2 | 0.000194818 |
| 3.3 | -0.051462421 |
| 3.4 | 0.027767494 |
| 3.5 | -0.024435394 |
| 3.6 | -0.047019622 |
| 3.7 | -0.038134024 |
| 3.8 | 0.034431692 |
| 4.1 | -0.069233617 |
| 4.2 | 0.014798641 |
| 4.3 | -0.036653091 |
| 4.4 | -0.040591567 |
| 4.5 | 0.018879799 |
| 4.6 | 0.022341095 |
| 4.7 | -0.001258653 |
| 4.8 | -0.023599748 |
| 5.1 | -0.054436753 |
| 5.2 | -0.026117055 |
| 5.3 | -0.062303335 |
| 5.4 | -0.034298301 |
| 5.5 | 0.000314663 |
| 5.6 | -0.00660793 |
| 5.7 | -0.073001888 |
| 5.8 | 0.031151668 |
| 6.1 | -0.042479547 |
| 6.2 | 0.015418502 |

| | |
|---|---|
| 6.3 | 0.011013216 |
| 6.4 | -0.052548773 |
| 6.5 | 0.004090623 |
| 6.6 | -0.082441787 |
| 6.7 | 0.015418502 |
| 6.8 | 0.002517306 |
| 7.1 | -0.082491997 |
| 7.2 | -0.114257572 |
| 7.3 | -0.029549372 |
| 7.4 | -0.04284659 |
| 7.5 | -0.038660428 |
| 7.6 | -0.168185176 |
| 7.7 | -0.220389067 |
| 7.8 | -0.11154888 |
| 8.1 | -0.02684068 |
| 8.2 | -0.186407289 |
| 8.3 | -0.028810638 |
| 8.4 | -0.102930313 |
| 8.5 | -0.001231224 |
| 8.6 | -0.063284905 |
| 8.7 | -0.060083723 |
| 8.8 | -0.088894361 |
| 9.1 | -0.010167268 |
| 9.2 | 0.02722204 |
| 9.3 | -0.013447032 |
| 9.4 | -0.022630371 |
| 9.5 | -0.028861922 |
| 9.6 | -0.074122663 |
| 9.7 | 0.022958347 |
| 9.8 | -0.026566087 |
| 9.9 | -0.04263693 |
| 9.10 | -0.0236143 |
| 9.11 | 0.020990489 |
| 9.12 | -0.052804198 |
| 9.13 | 0.02722204 |
| 9.14 | -0.004263693 |
| 9.15 | -0.017054772 |
| 9.16 | -0.017054772 |
| 9.17 | 0.007733692 |
| 9.18 | 0.008069939 |
| 9.19 | 0.001681237 |
| 10.1 | 0.03718296 |
| 10.2 | -0.111795124 |
| 10.3 | -0.061068702 |
| 10.4 | -0.035705491 |

| | |
|---|---|
| 10.5 | -0.049987688 |
| 10.6 | -0.093573012 |
| 10.7 | 0.045977011 |
| 10.8 | 0.049763567 |
| 11.1 | -0.033624748 |
| 11.2 | -0.00537996 |
| 11.3 | -0.146267653 |
| 11.4 | -0.009414929 |
| 11.5 | 0.089778077 |
| 11.6 | 0.006052455 |
| 11.7 | -0.014122394 |
| 11.8 | -0.003026227 |
| 11.9 | -0.099193006 |
| 11.10 | -0.037995965 |
| 11.11 | 0.059224625 |
| 11.12 | -0.007397445 |
| 11.13 | -0.014416521 |
| 11.14 | 0.043639197 |
| 11.15 | 0.019838601 |
| 11.16 | 0.013449899 |
| 11.17 | 0.020847344 |
| 11.18 | -0.04270343 |
| 11.19 | -0.032279758 |
| 12.1 | 0.011883889 |
| 12.2 | 0.059224625 |
| 12.3 | 0.058834989 |
| 12.4 | -0.000450349 |
| 12.5 | 0.03253458 |
| 12.6 | 0.027020941 |
| 12.7 | -0.024157413 |
| 12.8 | 0.102279369 |
| 13.1 | 0.064095071 |
| 13.2 | 0.034676875 |
| 13.3 | 0.047511822 |
| 13.4 | -0.026345418 |
| 13.5 | -0.052240486 |
| 13.6 | 0.0229678 |
| 13.7 | -0.000225175 |
| 13.8 | 0.045260077 |
| 14.1 | 0.0344517 |
| 14.2 | -0.03197478 |
| 14.3 | -0.009546074 |
| 14.4 | 0.040633894 |
| 14.5 | -0.015782665 |
| 14.6 | 0.009156439 |

| | |
|---|---|
| 14.7 | 0.011384217 |
| 14.8 | 0.045536869 |
| 15.1 | 0.033117723 |
| 15.2 | 0.0633158 |
| 15.3 | 0.04792519 |
| 15.4 | 0.070329242 |
| 15.5 | 0.030530401 |
| 15.6 | 0.040103493 |
| 15.7 | -0.001363725 |
| 15.8 | 0.048899279 |
| 16.1 | -0.001719198 |
| 16.2 | 0.000517464 |
| 16.3 | -0.010349288 |
| 16.4 | -0.008022923 |
| 16.5 | 0.013195343 |
| 16.6 | -0.025614489 |
| 16.7 | -0.027684347 |
| 16.8 | -0.002292264 |
| 17.1 | -0.000573066 |
| 17.2 | 0.019529294 |
| 17.3 | 0.017025538 |
| 17.4 | 0.020030045 |
| 17.5 | 0.027290936 |
| 17.6 | 0.017275914 |
| 17.7 | 0.013019529 |
| 17.8 | 0.009514271 |
| 18.1 | -0.02428643 |
| 18.2 | -0.025538307 |
| 18.3 | 0.048322484 |
| 18.4 | 0.006259389 |
| 18.5 | 0.028292439 |
| 18.6 | 0.00901352 |
| 18.7 | 0.044316475 |
| 18.8 | 0.070200573 |
| 19.1 | 0.024036054 |
| 19.2 | 0.016524787 |
| 19.3 | 0.01977967 |
| 19.4 | 0.009263896 |
| 19.5 | 0.013770656 |
| 19.6 | 0.015022534 |
| 19.7 | 0.014271407 |
| 19.8 | -0.047184509 |
| 22.1 | -0.014021812 |
| 22.2 | -0.03138215 |
| 22.3 | -0.040952593 |

| | |
|---|---|
| 22.4 | -0.007789895 |
| 22.5 | -0.102158914 |
| 22.6 | 0.009347874 |
| 22.7 | -0.047629646 |
| 22.8 | 0.020253728 |
| 23.1 | -0.035833519 |
| 23.2 | -0.02003116 |
| 23.3 | -0.039172045 |
| 23.4 | -0.008309456 |
| 23.5 | -0.046739372 |
| 23.6 | 0.013131538 |
| 23.7 | -0.083240596 |
| 23.8 | 0.032951289 |
| 24.1 | -0.056754952 |
| 24.2 | 0.03008596 |
| 24.3 | -0.050523036 |
| 24.5 | 0.004451369 |
| 24.6 | -0.07990207 |
| 24.7 | -0.058886224 |
| 24.8 | -0.048494538 |
| 24.9 | 0.016254246 |
| 25.1 | -0.018651745 |
| 25.2 | -0.070876632 |
| 25.3 | -0.042632561 |
| 25.4 | -0.111111111 |
| 25.5 | 0.027178257 |
| 25.6 | -0.098321343 |
| 25.7 | -0.063682387 |
| 25.8 | -0.052224887 |
| 26.1 | -0.05249134 |
| 26.2 | -0.054356515 |
| 26.3 | -0.038102851 |
| 26.4 | -0.079669598 |
| 26.5 | -0.083399947 |
| 26.6 | 0.000799361 |
| 26.7 | -0.067412736 |
| 26.8 | -0.06314948 |
| 27.1 | -0.077005063 |
| 27.2 | -0.030375699 |
| 27.3 | -0.028776978 |
| 27.4 | -0.038635758 |
| 27.5 | 0.008022923 |
| 27.6 | 0.040627227 |
| 27.7 | 0.00974212 |
| 27.8 | -0.094084105 |

| | |
|---|---|
| 28.1 | -0.049536707 |
| 28.2 | 0.038132573 |
| 28.4 | -0.013753582 |
| 28.5 | -0.092658589 |
| 28.6 | 0.017106201 |
| 28.7 | -0.005345688 |
| 28.8 | 0.029579473 |
| 28.12 | 0.034093123 |
| 29.1 | -0.045260157 |
| 29.2 | 0.012893983 |
| 29.3 | 0.041211689 |
| 29.4 | -0.115823236 |
| 29.5 | 0.031004989 |
| 29.6 | -0.002292264 |
| 29.7 | -0.027084818 |
| 29.8 | -0.028153956 |
| 30.1 | 0.017818959 |
| 30.2 | -0.025302922 |
| 30.3 | 0.03492516 |
| 30.4 | -0.020057307 |
| 30.5 | 0.006017192 |
| 30.6 | 0.017191977 |
| 30.7 | 0.039509537 |
| 30.8 | 0.009169054 |
| 31.1 | -0.030659026 |
| 31.2 | -0.001432665 |
| 31.3 | 0.040054496 |
| 31.4 | -0.001146132 |
| 31.5 | 0.008022923 |
| 31.6 | -0.005730659 |
| 31.7 | 0.01852861 |
| 31.8 | 0.087921348 |
| 32.1 | 0.028610354 |
| 32.2 | 0.017134831 |
| 32.3 | 0.033426966 |
| 32.4 | 0.014713896 |
| 32.5 | 0.014044944 |
| 32.6 | 0.050561798 |
| 32.7 | 0.004494382 |
| 32.8 | 0.070786517 |
| 33.1 | 0.016893733 |
| 33.2 | 0 |
| 33.3 | -0.004455446 |
| 33.4 | 0.098033708 |
| 33.5 | -0.07549505 |

| | |
|---|---|
| 33.6 | 0.030898876 |
| 33.7 | -0.057920792 |
| 33.8 | 0.071067416 |
| 34.1 | 0.033415842 |
| 34.2 | 0.023514851 |
| 34.3 | 0.014356436 |
| 34.4 | -0.089356436 |
| 34.5 | -0.071782178 |
| 34.6 | 0.044059406 |
| 34.7 | 0.018316832 |
| 34.8 | 0.020544554 |
| 35.1 | -0.108168317 |
| 35.2 | 0.025280899 |
| 35.3 | -0.062871287 |
| 35.4 | 0.02549505 |
| 35.5 | 0.015841584 |
| 35.6 | 0.040594059 |
| 35.7 | -0.044059406 |
| 35.8 | 0.006502242 |
| 36.1 | -0.002466368 |
| 36.2 | 0.006278027 |
| 36.3 | 0.020852018 |
| 36.4 | 0.046188341 |
| 36.5 | 0.018834081 |
| 36.6 | 0.010986547 |
| 36.7 | 0.035874439 |
| 36.8 | -0.002690583 |
| 37.1 | 0.019058296 |
| 37.2 | 0.017713004 |
| 37.3 | 0.024215247 |
| 37.4 | 0.010538117 |
| 37.5 | 0.024439462 |
| 37.6 | 0.017264574 |
| 37.7 | 0.015919283 |
| 37.8 | 0.020179372 |
| 38.1 | 0.026233184 |
| 38.2 | 0.011659193 |
| 38.3 | 0.027578475 |
| 38.4 | 0.025336323 |
| 38.5 | 0.008071749 |
| 38.6 | 0.058988764 |
| 38.8 | 0.013483146 |
| 39.2 | 0.114606742 |
| 39.3 | -0.016853933 |
| 39.4 | 0.061797753 |

| | |
|---|---|
| 39.5 | -0.018258427 |
| 39.6 | 0.076404494 |
| 39.7 | 0.075561798 |
| 39.8 | -0.014212277 |
| 40.1 | 0.045302946 |
| 40.2 | -0.140913214 |
| 40.3 | 0.040374962 |
| 40.4 | -0.004233444 |
| 40.5 | 0.104021772 |
| 40.6 | 0.037193831 |
| 40.7 | -0.019352888 |
| 40.8 | 0.178710395 |
| 41.1 | 0.000277932 |
| 41.2 | 0.032960387 |
| 41.3 | -0.001111729 |
| 41.4 | 0.048986997 |
| 41.5 | 0.039093041 |
| 41.6 | 0 |
| 41.7 | 0.012770394 |
| 41.8 | 0.021631483 |
| 42.1 | -0.015897837 |
| 42.2 | 0.043002346 |
| 42.3 | 0.008079229 |
| 42.4 | -0.023716445 |
| 42.5 | 0.026583268 |
| 42.6 | 0.004691165 |
| 42.7 | 0.09918355 |
| 42.8 | 0.013031014 |
| 43.1 | 0.250982764 |
| 43.2 | 0.045608548 |
| 43.3 | -0.008600469 |
| 43.4 | 0.032577535 |
| 43.5 | 0.150589658 |
| 43.6 | 0.032577535 |
| 43.7 | 0.087995162 |
| 43.8 | -0.022673964 |
| 44.1 | 0.117024493 |
| 44.2 | 0.01770985 |
| 44.3 | -0.00727802 |
| 44.4 | -0.005745389 |
| 44.5 | -0.014798641 |
| 44.6 | 0.059872997 |
| 44.7 | 0.011159631 |
| 44.8 | 0.04269772 |
| 45.1 | 0.015041242 |

| | |
|---|---|
| 45.2 | 0.108557605 |
| 45.3 | 0.023774867 |
| 45.4 | 0.043910723 |
| 45.5 | 0.025230471 |
| 45.6 | 0.046265497 |
| 45.7 | 0.045366327 |
| 45.8 | -0.013909888 |

Supplemental Table S2. The relationship between the populations of heads of cattle, sheep, and goats reared within specific municipalities of the Central-eastern mesoregion of Paraná state, Brazil in 2019.

| Municipalities | Livestock populations [1] | | | Ratios | |
|---|---|---|---|---|---|
| | Cattle | Goats | Sheep | Sheep: cattle | Goat: cattle |
| Arapoti | 48,197 | 134 | 2,188 | 0.05 | 0.00 |
| Jaguariaíva | 28,158 | 224 | 3,468 | 0.12 | 0.01 |
| Carambeí | 49,979 | 120 | 1,589 | 0.03 | 0.00 |
| Castro | 120,674 | 605 | 14,532 | 0.12 | 0.01 |
| Palmeira | 25,638 | 374 | 8,168 | 0.32 | 0.01 |

1. IBGE. Pesquisa da Pecuária Municipal [Municipal Livestock Research]. Available online: https://www.ibge.gov.br/estatisticas/economicas/agricultura-e-pecuaria/9107-producao-da-pecuaria-municipal.html?=&t=resultados (accessed on 19 March 2024)
